# Challenging the Paradigm: Anti-Inflammatory Interleukins and Angiogenesis

**DOI:** 10.3390/cells11030587

**Published:** 2022-02-08

**Authors:** Amanda M. Peluzzo, Michael V. Autieri

**Affiliations:** Lemole Center for Integrated Lymphatics Research, Department of Cardiovascular Sciences, Lewis Katz School of Medicine at Temple University, Philadelphia, PA 19140, USA; amanda.peluzzo@temple.edu

**Keywords:** inflammation, hypoxia, cytokine, interleukin, angiogenesis, endothelial cell, macrophage, polarization

## Abstract

Angiogenesis is a vital biological process, and neovascularization is essential for the development, wound repair, and perfusion of ischemic tissue. Neovascularization and inflammation are independent biological processes that are linked in response to injury and ischemia. While clear that pro-inflammatory factors drive angiogenesis, the role of anti-inflammatory interleukins in angiogenesis remains less defined. An interleukin with anti-inflammatory yet pro-angiogenic effects would hold great promise as a therapeutic modality to treat many disease states where inflammation needs to be limited, but revascularization and reperfusion still need to be supported. As immune modulators, interleukins can polarize macrophages to a pro-angiogenic and reparative phenotype, which indirectly influences angiogenesis. Interleukins could also potentially directly induce angiogenesis by binding and activating its receptor on endothelial cells. Although a great deal of attention is given to the negative effects of pro-inflammatory interleukins, less is described concerning the potential protective effects of anti-inflammatory interleukins on various disease processes. To focus this review, we will consider IL-4, IL-10, IL-13, IL-19, and IL-33 to be anti-inflammatory interleukins, all of which have recognized immunomodulatory effects. This review will summarize current research concerning anti-inflammatory interleukins as potential drivers of direct and indirect angiogenesis, emphasizing their role in future therapeutics.

## 1. Introduction

Sprouting angiogenesis is the expansion of new blood vessels from pre-existing vasculature where tip cells are stimulated to express proteases, migrate, proliferate, and form new tubes [1,2]. This is distinct from vasculogenesis which is the de novo formation of blood vessels via differentiation of circulating precursor cells, known as angioblasts, to endothelial cells [3,4]. In addition, intussusceptive angiogenesis is distinct from both sprouting angiogenesis and vasculogenesis. Intussusceptive angiogenesis occurs when the vascular wall of an existing blood vessel invaginates and forms a pillar, splitting one blood vessel into two [5,6,7]. Regardless of the type of angiogenesis, the goal is to increase blood vessel availability to hypoxic or expanding tissue mass, ensuring the metabolic demands of the tissue are met. Due to the diffusion limit for oxygen, a cell is no further than 100–200 μm from a capillary [1,2].

Throughout embryonic and postnatal development, the body size rapidly increases and inexorably experiences an immense amount of angiogenesis [1,8,9]. Physiological angiogenesis must also occur in healthy tissue throughout adult life to meet the basic functional demands of each tissue. For example, exercise stimulates angiogenesis in cardiac and skeletal muscle [10,11]. Neovascularization is also a necessary vascular expansion-based repair mechanism to preserve tissue viability in response to ischemic conditions. Reparative angiogenesis occurs as an attempt to restore tissue perfusion to salvage tissue after hypoxia, as seen in ischemic cardiomyopathy and peripheral arterial disease. On the other hand, pathological angiogenesis is often the target of anti-tumor therapy as it contributes to tumor growth and metastasis [1,12]. Tissue hypoxia initiates both inflammatory and angiogenic factors to promote angiogenesis in an attempt to restore perfusion, and, as with any complex process, revascularization of ischemic tissue involves multiple cell types. While neovascularization and inflammation are independent biological processes, they are linked in response to injury and ischemia, and both pro-inflammatory and anti-inflammatory processes participate in angiogenesis. Better identification of the soluble autocrine and paracrine factors which participate in these processes will increase our understanding of reparative angiogenesis and lead to more effective therapeutic modalities which resolve inflammation while maintaining tissue perfusion. The purpose of this review is to describe such soluble factors.

In terms of tissue repair, the paradigm of cytokine function in relation to angiogenesis loosely adheres to the Janus Phenomenon first described by Epstein et al. in 2004. This states that, in general, anti-inflammatory cytokines are anti-angiogenic while pro-inflammatory cytokines are pro-angiogenic [13,14]. While clear that pro-inflammatory factors drive angiogenesis, the role of anti-inflammatory cytokines in angiogenesis remains uncertain. With these limitations in mind, we do recognize that some anti-inflammatory cytokines are indirectly pro-angiogenic through macrophage polarization toward the alternative M2 phenotype, which secretes vascular endothelial growth factors (VEGFs) [15]. While intuitive that the inflammatory state of ischemic tissue may dictate whether that tissue becomes neovascularized or necrotic, the participation of indirect effects of anti-inflammatory interleukins on the initiation of angiogenesis remains largely uncharacterized.

**Figure 1:**
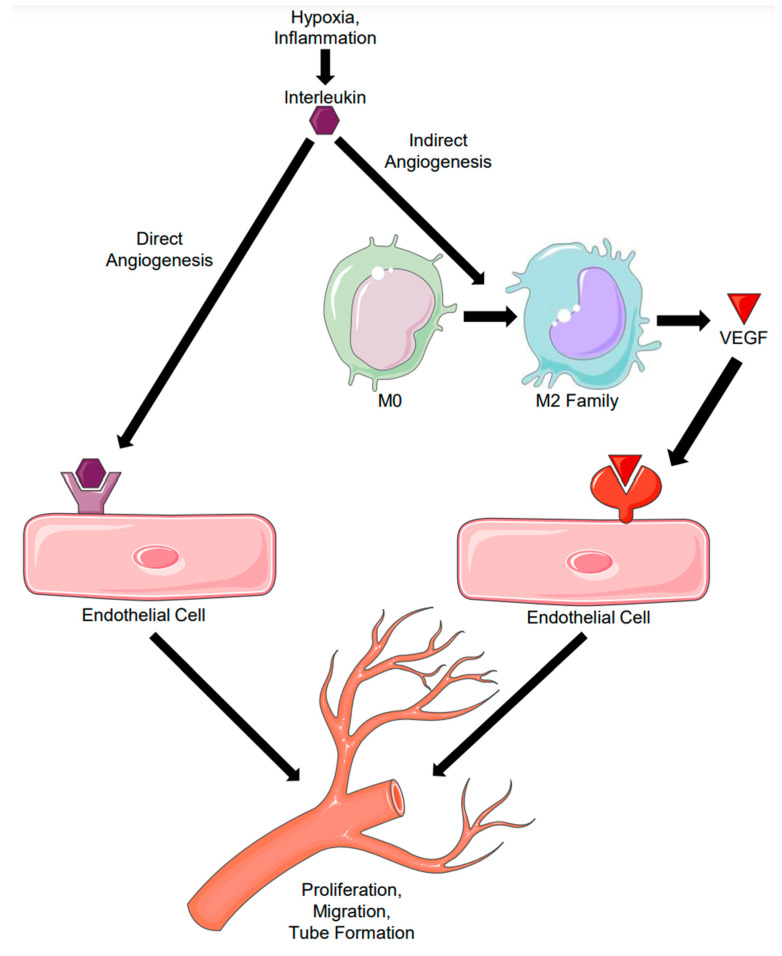
Direct versus Indirect Angiogenesis. Direct angiogenesis results from an interleukin binding directly to its receptor present on an endothelial cell, leading to the induction of angiogenesis. Indirect angiogenesis results from an interleukin inducing macrophage polarization and subsequent VEGF secretion, leading to the same endpoint of angiogenesis.

**Figure 2:**
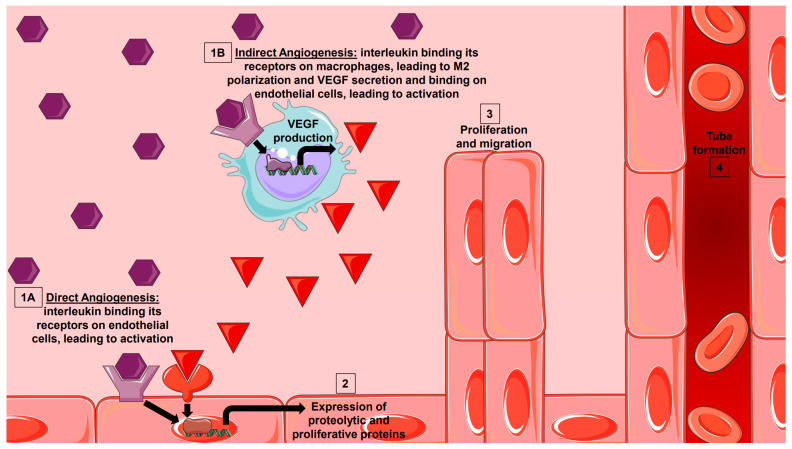
Interleukin Initiation of Sprouting Angiogenesis. Interleukins are secreted from various effector cells in health and disease. They can either bind to their receptor directly on endothelial cells (1A) to induce expression of proteolytic and proliferative factors, or they can indirectly bind to their receptor on macrophages (1B) to induce M2 family polarization and VEGF production. VEGF can then bind to its receptor on endothelial cells to induce the expression of proteolytic and proliferative factors. Both direct and indirect angiogenesis can lead to proliferation, migration, and tube formation seen in sprouting angiogenesis (3, 4).

For the purposes of this review, we will classify cytokine-mediated angiogenesis as “direct”, meaning, the response of endothelial cells to the cytokine results in new vessel formation via sprouting angiogenesis, or “indirect”, meaning cytokine-induced macrophage polarization to the M2 phenotype and subsequent release of factors from that macrophage induces endothelial cell angiogenesis (Figure 1 and Figure 2).

## 2. Direct Angiogenesis: Molecular Mechanisms of Endothelial-Induced Angiogenesis

The overarching mechanism which drives hypoxia-induced angiogenesis relies on the regulation of hypoxia-inducible factor-1 (HIF-1), a transcription factor that induces angiogenic gene expression [16]. HIF-1 is a heterodimer composed of HIF-1α and HIF-1β, with dimerization mediated by prolyl hydroxylase domain-containing enzymes (PHDs) [16,17]. Under normoxic conditions, PHDs utilize oxygen and 2-oxoglutarate to hydroxylate central prolines within HIF-1α, which are then recognized by the von Hippel–Lindau tumor suppressor protein (pVHL), leading to polyubiquitination and proteasomal degradation of HIF-1α [18]. In this way, low levels of HIF-1α ensure minimal angiogenesis under normoxic conditions. In hypoxic conditions, there is less oxygen co-substrate available for PHDs, promoting HIF-1α stability and allowing dimerization with HIF-1β. The dimer translocates to the nucleus, where it transactivates the expression of numerous angiogenic genes ranging from VEGFs to chemokines [16,18]. The endothelial cell (EC) response to hypoxia is loosely considered to a six-step process that leads to sprouting angiogenesis, briefly outlined as follows: (1) HIF1α activation in response to hypoxia initiates expression of angiogenesis-related genes; (2) expression of proteases by capillary tip cells enables ECs to migrate through the basal lamina of the parent vein or capillary; (3) migration toward the chemokine gradient which is greatest at the hypoxic region; (4) proliferation of ECs to generate sufficient cells to form a new conduit; (5) differentiation of the ECs to form a tube-like structure; (6) investment of pericytes around the newly formed vessel to stabilize it. As the new vessel forms, blood is brought to the tissue and oxygen concentration rises, leading to reduced HIF-1α stability and activity, thus ending the angiogenic process [10]. 

## 3. Indirect Angiogenesis: Macrophage Polarization-Induced Angiogenesis

Macrophages exhibit functional plasticity in which they can alter their phenotype in response to environmental cues, allowing them to better modify their response to proximal stimuli and exert specific functions depending on the nearby signals. Monocytes differentiate into non-activated macrophages, which are then considered M0. Although less clearly delineated in humans, activated macrophages in mice are generally divided into two major populations: M1 or M2. Classically activated or M1 macrophages are induced by pro-inflammatory stimuli such as LPS and Th1 cytokines (IFNγ and TNFα) [19]. It was found that macrophage activation by Th2 cytokines, such as IL-4 or IL-13, leads to an alternatively activated M2 macrophage [19]. M1 macrophages are associated with inflammation, while M2 macrophages are associated with wound healing and neovascularization [20]. 

Within M2 macrophages, subtypes of M2a, M2b, M2c, and M2d were identified. These macrophage subtypes differ in various cell surface and genetic markers, which are used for classification purposes, but identification is not as clearly defined in vivo, which makes it difficult to study and translate to human disease. All M2 subtypes are stimulated by different factors, but for the purpose of this review, we will focus on the anti-inflammatory cytokines listed below and their ability to polarize macrophages toward a given subtype. Each M2 subtype also varies in its functionality. M2a macrophages function through reparative wound healing via tissue remodeling with anti-inflammatory and pro-angiogenic effects [19]. M2b macrophages are immunomodulatory and secrete both pro-inflammatory (IL-1β, TNFα, IL-6) and anti-inflammatory cytokines (IL-10, IL-12) in response to immune complexes [19,21]. M2c macrophages perform immunologically silent phagocytosis known as efferocytosis and are major effectors in tissue remodeling, which is why they are often referred to as regulatory macrophages [19,22,23]. M2d macrophages, also called tumor-associated macrophages (TAMs), contribute to tumor growth by promoting neovascularization and allowing the tumor to prosper [19,24]. 

To further confound classification, many in vivo studies point to the presence of “hybrid” macrophages that display more than one subtype marker [25]. With this in mind, it is important to scrupulously study and understand macrophage polarization and their subsequent contribution to disease promotion or resolution, as well as their contribution to angiogenesis. Nevertheless, most investigations suggest a more prominent role of M2 macrophages in angiogenesis by virtue of the repertoire of their pro-angiogenic secretome [26]. M2 macrophages express a significant number of soluble angiogenic factors such as VEGF-A, TGFα, and PDGF-β, which act in a paracrine manner to promote wound repair and neovascularization on local endothelial cells [20,22]. These macrophage-derived cytokines can elicit the pro-angiogenic processes on EC outlined in the previous section. This is best illustrated by the observation that conditioned media from M2 macrophages induce angiogenic responses in EC, while conditioned media from M0 and M1 macrophages inhibit angiogenic responses in EC [19,20]. Thus, cytokines that polarize macrophages to the M2 phenotype can be considered indirectly angiogenic by virtue of their ability to elicit angiogenic cytokine synthesis from those macrophages.

## 4. Anti-Inflammatory Cytokines 

The molecular mechanisms of immune modulation by anti-inflammatory cytokines are numerous and diverse. Most studies of their effects are performed with immune cells. Many of these effects are mediated through signaling cascades involving the JAK and STAT signaling proteins, synthesis of the suppressor of cytokine signaling (SOCS) family proteins, and modulation of NF-κB activity [27,28,29].

A great deal of attention is given to the negative effects of pro-inflammatory interleukins in tissue ischemia, but far less is described concerning the capacity of anti-inflammatory interleukins to promote wound healing and angiogenesis. To focus this review, we will consider IL-4, IL-10, IL-13, IL-19, and IL-33 as anti-inflammatory interleukins, and we will describe their effects on macrophage polarity and effects on endothelial cells, both contributing to their angiogenic potential. It is important to note that, although these interleukins have angiogenic properties, the literature is complex and, at times, contradictory. These contradictory conclusions are often drawn from studying an interleukin in a particular disease model before determining its basic effects on endothelial cells and its role in physiology. Therefore, future studies should focus on rigorous analysis of the direct and indirect angiogenic effects both in vitro and in vivo prior to interpretation within the context of a complex disease model. Our aim is to present a balanced picture of the angiogenic potential of these interleukins, including both pro- and anti-angiogenic reports.

Interleukin-4 (IL-4) is an anti-inflammatory interleukin recognized to polarize T cells to their Th2 phenotype. IL-4 is secreted by CD4+ T-cells, NK T-cells, basophils, eosinophils, mast cells, and type 2 innate lymphoid cells [30]. Earlier studies by Volpert et al. indicated that IL-4 is an inhibitor of angiogenesis both in vitro and in vivo as an anti-tumor agent [31]. Although, at lower IL-4 treatment concentrations, neovascularization was induced, indicating possible angiogenic potential that is concentration-dependent [31]. IL-4 is characterized for its involvement in chronic inflammatory lung diseases such as asthma, and one study analyzed pulmonary angiogenesis under hypoxic conditions in an IL-4 knockout (KO) model [32]. This study concluded that, although VEGF-A was upregulated, pulmonary angiogenesis was diminished in the absence of IL-4, confounding the role of IL-4 in hypoxia-induced angiogenesis from earlier reports [32]. Later studies have also indicated that IL-4 promotes angiogenesis via direct effects on endothelial cells [33]. One such study focused on age-related macular degeneration and concluded that IL-4 promoted choroidal neovascularization both by directly stimulating tube formation in endothelial progenitor cells and by inducing vasculogenesis through bone marrow-derived endothelial progenitor cells [33]. Another study in an atopic dermatitis model showed that IL-4 dysregulates the expression of microRNAs involved in angiogenesis which may contribute to the excessive angiogenesis seen in disease pathogenesis [34]. Other studies have indicated IL-4 as a potent mitogen that induces tube formation directly in vitro and in vivo, again contradicting the earlier studies by Volpert et al. [35,36,37]. IL-4 also polarizes macrophages toward the alternative M2a phenotype, increasing its angiogenic potential through indirect angiogenesis [19,20]. The summation of these studies points toward IL-4 as a pro-angiogenic interleukin albeit with ill-defined mechanisms.

Interleukin-10 (IL-10) is the archetypical anti-inflammatory interleukin and consequently the most studied. IL-10 is recognized to attenuate the expression of inflammatory transcripts in multiple cell types and inhibit antigen presentation and T-cell proliferation [38]. IL-10 is produced by CD4+ T-cells, CD8+ T-cells, NK T-cells, mast cells, neutrophils, eosinophils, monocytes, macrophages, dendritic cells, and under certain circumstances, B-cells [39]. Typically, IL-10 is released in response to pathogen-associated molecular patterns that are recognized by toll-like receptors in areas of inflammation [39]. Very few studies have focused on the direct angiogenic potential IL-10 may have on endothelial cells. One study indicated that IL-10 had anti-tumor and anti-angiogenic effects [40]. They performed a dorsal air sac assay using human ovarian cell lines transferred with murine IL-10 plasmid and revealed decreased microvessel formation in the presence of IL-10, although VEGF levels remained unaffected [40]. Another study indicated IL-10’s anti-angiogenic effect using the hind limb ischemia mouse model and revealed increased angiogenesis in an IL-10 KO mouse model with a subsequent decrease in angiogenesis when treated with murine IL-10 plasmid [41]. To date, no mechanistic studies were reported to characterize the direct effect of IL-10 on endothelial cells in vitro. On the other hand, one study, which specifically focused on the regulation of IL-10 in B-cells, indicated that HIF-1α has response elements in the IL-10 promoter, resulting in the expression of IL-10 under hypoxic conditions perhaps suggesting an indirect angiogenic function [42]. IL-10 is a recognized M2 polarizing cytokine [19,20]. In an intraocular neovascularization model focusing on IL-10’s role in macrophage polarization, IL-10 was implicated as being indirectly angiogenic since ischemia-induced pathological angiogenesis in the retina was promoted in the presence of IL-10 [43]. Later studies determined that IL-10 specifically polarizes macrophage to the M2c phenotype, suggesting a role in wound healing and tissue remodeling [19,20]. In addition, M2c transcriptomic analysis revealed IL-10 induced the upregulation of angiogenic genes, corresponding to prior studies that indicated the angiogenic abilities of the M2c phenotype [44]. When taken together, these studies suggest that, while IL-10 has no direct angiogenic effects on EC to date, it does have the potential to be pro-angiogenic by virtue of its ability to modulate macrophages to the reparative phenotype.

Interleukin-13 (IL-13) is closely related to IL-4 and is similarly secreted by CD4+ T-cells, NK T-cells, basophils, eosinophils, mast cells, and type 2 innate lymphoid cells [30]. However, IL-13 differs from IL-4 in its receptor specificity [30]. Interestingly, despite this difference, IL-13 has many biological properties similar to IL-4, one example being the polarization of macrophages to the M2a phenotype, with subsequent expression of angiogenic cytokines, providing IL-13 with indirect angiogenic potential [19,30]. The combination of IL-13 with IL-4 potently polarizes macrophages to an anti-inflammatory phenotype [19,30]. That said, the effect of IL-13 on angiogenesis is controversial. Limited studies suggest that IL-13 plays a role in direct angiogenesis by promoting endothelial cell migration [45]. Other studies also suggest it is pro-angiogenic via the induction of tube formation in vitro and neovascularization of rat corneas in vivo [37]. Like IL-4, there is conflicting data where studies also suggest IL-13 attenuates vascular tube formation [46]. For example, physiologic concentrations of IL-13 also demonstrate anti-lymphangiogenic effects and potently impair lymphatic endothelial survival, proliferation, migration, and tube formation [47]. Inhibition of IL-13 activity by neutralizing antibodies also promotes lymphangiogenesis [47]. Clearly, a more thorough analysis of angiogenic assays both on cultured EC and mouse models are required to make a definitive statement on IL-13’s association with angiogenesis. 

Interleukin-19 (IL-19) was originally placed in an IL-10 sub-family where the original “IL-10-related” cytokines (IL-20, IL-22, IL-24, IL-26, IL-29, as well as IL-19) were grouped together without any knowledge of their biological function but based solely on their amino acid similarly [48]. IL-19 is now considered to be in a sub-family that includes IL-20, IL-22, and IL-24. IL-19 is functionally distinct from the other sub-family members in terms of cell-specific expression, receptor usage, and function [49,50,51,52]. IL-19 is immunomodulatory, more specifically, anti-inflammatory [53,54,55]. Basal levels of IL-19 are shown to be secreted by keratinocytes, epithelial cells, macrophages, and to a lesser extent by activated B-cells [56]. However, unlike other family members, IL-19 expression can be induced by pro-inflammatory stimuli in ECs and macrophages [57,58]. Serum IL-19 levels have been shown to increase in various inflammatory disorders, such as psoriasis and inflammatory bowel disease, in both humans and mice, likely as a compensatory event to resolve inflammation [59,60,61]. Although retrospective in design, one human study examined the levels of HIF-1α and IL-19 in chronic obstructive pulmonary disease progression and identified an increase in the levels of both factors that were associated with disease progression [62]. IL-19 expression is induced in hind limb ischemia models [63]. Injection of recombinant mouse IL-19 increased hind limb perfusion and capillary density through directly increasing angiogenic gene expression in ECs as well as indirectly through macrophage polarization toward the M2 phenotype [57,63,64]. The absence of IL-19 by genetic deletion decreased perfusion and capillary density in the hind limb, though the effects could not be determined to be by deletion in macrophages or endothelial cells [63]. In cultured EC, IL-19 increases proliferation, migration, and tube formation. Mechanistically, at least in cultured EC, IL-19 induces angiogenesis by induction of RNA-binding protein ILF3, which increases mRNA stability of angiogenic cytokines [63,65]. When taken together, IL-19 may be the prime example of an exception to the Janus phenomenon, having anti-inflammatory, yet pro-angiogenic properties, by both direct effects on EC and indirect effects by macrophage polarization.

Interleukin-33 (IL-33) is an IL-1 family member and is expressed by macrophages, dendritic cells, mast cells, fibroblasts, osteoblasts, endothelial cells, and damaged epithelial cells [66,67]. It polarizes leukocytes to Th2 and macrophage to the M2 phenotype by induction of IL-4, IL-5, and IL-13 while also decreasing expression of IFNγ [68]. Its expression in human rheumatoid arthritis patients determined the presence of a regulatory circuit consisting of HIF-1α and IL-33, leading to the preservation of disease pathogenesis and indicating that IL-33 expression is induced by hypoxia to maintain hypoxic gene activation [69]. A more causal role of this cytokine during inflammation is complicated, and it depends on the disease process as well as the specific tissues involved. One review summarized IL-33 as being either pro- or anti-inflammatory, which varied by disease model [70]. For example, IL-33 exacerbated a murine model of collagen-induced arthritis and increased the generation of pro-inflammatory cytokines [71]. IL-33 is also implicated in the disease phenotypes of asthma, atopic dermatitis, and psoriasis [70]. Anti-IL-33 antibody treatment was shown to prevent the negative histological adaptations commonly associated with asthma in an OVA-induced asthma mouse model [72]. On the other hand, IL-33 is proposed to be protective against obesity and type 2 diabetes by increasing Th2 cytokines and inducing M2 macrophage polarization [70]. This divergence of IL-33’s actions further emphasizes the importance of critically analyzing each cytokine’s possible mechanisms of action on a tissue of interest before introducing it to a disease model. That said, the IL-33 receptor and its cognate receptor ST2 are expressed on EC, and studies in cultured EC have shown that IL-33 increases direct angiogenesis via EC proliferation, migration, and tube formation, which was prevented by knockdown of ST2 [73]. IL-33 also polarizes macrophages toward the alternatively activated M2 phenotype, and limited studies suggest M2a subtype involvement [74]. 

In summary, analysis of the expression of anti-inflammatory interleukins and their effects on both endothelial cells and macrophages are vital to the true understanding of their role in direct and indirect angiogenesis, respectively. At present, this is challenging, as most studies focus on disease processes in vivo without performing classical sprouting angiogenesis assays such as proliferation, migration, and tube formation in vitro on cultured endothelial cells, making it difficult to distinguish direct from indirect angiogenic effects. Additional limitations include the type of angiogenesis being analyzed. As mentioned in the introduction, there are multiple ways a blood vessel can be formed which includes, but is not limited to, sprouting angiogenesis, vasculogenesis, and intussusceptive angiogenesis. For the sake of simplicity, this review focused on sprouting angiogenesis, but studies should also include analysis of the effects on vasculogenesis and intussusceptive angiogenesis. This would require a more thorough analysis of emerging in vitro and in vivo assays [6,75]. Future studies should begin in vitro, determining the binding and activation of endothelial cells by cytokines. Further in vitro experiments can determine macrophage activation by cytokines and subsequent secretion of VEGFs. Then, transitioning to in vivo experiments would elucidate the functionality in a dynamic system. Finally, the introduction of disease models can be introduced to determine if perturbations are seen.

Further clarification of the molecular mechanisms of these angiogenic effects is needed to identify protein participants of these angiogenic pathways, as each represents a target of rational drug therapy. What is recognized, however, is the promise that anti-inflammatory interleukins hold as effectors of wound healing. A greater understanding of these interleukins, their angiogenic effects, and the molecular mechanisms behind them will bolster their use in multiple diseases and emphasize their role in translational medicine. Table 1 provides a review of the above information, organized by cytokine.

## Figures and Tables

**Table 1:** Characterization of anti-inflammatory cytokines.

Cytokine	Macrophage Phenotype Induced	Direct Angiogenic Potential
IL-4	M2a [19,20]	Controversial [33,34,35,36,37]
IL-10	M2c [19,20]	Controversial [40,41,42,43]
IL-13	M2a [19,30]	Controversial [37,45,46,47]
IL-19	M2a [57,63]	Yes [58,63,64,65]
IL-33	M2a [19,74]	Yes [73]
